# Age Estimation in Children by the Measurement of Open Apices in Teeth: A Study in the Western Indian Population

**DOI:** 10.1155/2022/9513501

**Published:** 2022-01-30

**Authors:** Sanjana Manjrekar, Shantanu Deshpande, Farhin Katge, Romi Jain, Tejaswini Ghorpade

**Affiliations:** ^1^Department of Pediatric and Preventive Dentistry, Terna Dental College, Navi Mumbai 400706, Maharashtra, India; ^2^Department of Public Health Dentistry, Terna Dental College, Navi Mumbai 400706, Maharashtra, India

## Abstract

In forensic sphere and clinical dentistry, age estimation is a topic of utmost importance. Various techniques are employed in children to determine age; however, dental development has proven to be an appropriate method because of its low variability. Cameriere's method is a widely accepted method of age estimation in children, which is carried out by measuring the projections of open apices and also the heights of developing permanent teeth seen on panoramic radiographs. The aim of this study is to establish a new formula for age estimation in the Western Indian population by measuring the open apices of mandibular teeth using Cameriere's European formula. For this study, we included 311 panoramic radiographs of healthy children living in Western India (Maharashtra, Gujarat, and Goa) aged 4–15 years which were analysed by two independent researchers. Seven left permanent mandibular teeth were assessed for length and width of open apices. Dental maturity was evaluated using measurements of the left seven permanent mandibular teeth (*x*_*i*_ = *A*_*i*_/*L*_*i*_, *i* = 1,…, 7), the sum of the normalized open apices (*s*), and the number (*N*0) of teeth with complete root formation. A linear relationship between open apices, *N*0, age, and other factors was evaluated with the aid of a stepwise multiple regression model. A stepwise linear regression showed that all parameters, gender, *s*, *N*0, and *x*_5_, were significantly associated with age (*R* = 85%). No statistically significant difference was found between the predicted and actual chronological age of children in the age group of 4–13 years using the regression equation for the Western Indian population. The present research suggests that the new regression formula developed will be more accurate for age assessment in the Western Indian population.

## 1. Introduction

Age estimation is one of the most remarkable contributions of forensic odontology for the identification of living and deceased humans. In forensic sphere, subject of age assessment is as old as the study of forensic anthropology. It has been conventionally associated with the analysis of skeletonized human remains which forms a part of biological profile estimation. Lately, there has been a paradigm shift towards age determination of living individuals. Age assessment has a lawful and humanitarian background since, most of the times, it is associated with age estimation of an individual in different scenarios [1, 2], in situations where subjects fail to provide identification documents in cases of adoption, unaccompanied minors seeking asylum, illegal cross-border immigration, and other civil matters [2, 3]. Age determination plays a vital role in pediatric dentistry and orthodontics for diagnosis and treatment planning. Radiological analysis for the skeletal development of hand-wrist bones, cervical vertebrae, and teeth is a commonly employed method for biological age assessment in children and adolescents [4]. Moreover, a single exam currently allows for a 360-degree evaluation of orthodontic and development problems, thanks to the new 3D radiographic information management and rendering software [5].

The development of an individual refers to biological-physiological age. This is not always in consistency with the chronological or the calendar age. As an individual grows, this gap between chronological age and physiological age widens under the influence of hormonal and racial factors, environmental changes, and various diseases. The age assessment methods are developed by taking into account factors that aim at analysing least affected tissues and organs [6]. Teeth are useful indicators of age in the subadult age group up to age of 16, principally because of their high heritability, a low coefficient of variation, more resistance to the influence of environmental factors, and lack of other reliable predictors [7–9].

Amongst several radiographic approaches of dental age determination, most widely used are Demirjian's, Nolla's, and Cameriere's method of age estimation. Nolla in 1960 proposed a method of age estimation by studying the mineralization status of permanent dentition in American children [10]. Demirjian et al. developed a scoring system based on the calcification of seven permanent mandibular teeth [11]. Although the Demirjian system has gained acceptance worldwide, studies testing applicability of this method in the Indian population revealed an overestimation of age, thus concluding that it cannot be used in Indian samples [12, 13]. Cameriere et al., in 2006, proposed a method of estimating chronological age in Italian children by deriving a linear regression formula. For determining dental age, radiographic width of open apices and length of the tooth for the first seven mandibular teeth were measured in this method [14]. Following this, Rai et al., in their study, evaluated dental age using Cameriere's European formula for the Indian population which failed to yield correct value. Hence, a new formula was proposed for the Indian population considering the differences between Central, North, and South Indian populations [15].

As the formula cannot be applied to the Western Indian population, the aim of this paper was to develop a population-specific formula for estimating age in children of the Western Indian population based on the method described by Cameriere et al. It also aims at determining the accuracy of this Western Indian-specific formula.

## 2. Materials and Methods

This retrospective research was conducted on panoramic radiographs of healthy children living in Western India (Maharashtra, Gujarat, and Goa) aged 4–15 years. Panoramic radiographs routinely recorded between 2018 and 2020 for diagnostic and therapeutic purposes were included. Radiographs were assessed using Cameriere's method wherein measurements of seven left permanent teeth were used for age assessment. The ethical clearance was obtained from the institutional review board.

### 2.1. Inclusion Criteria and Exclusion Criteria

The inclusion criteria were panoramic radiographs of healthy patients in the age group of 4–15 years. Panoramic radiographs with proper exposure, maximum contrast, optimum density, and exact anatomical representation were included. Radiographs showing at least a single tooth with incomplete root formation among the first seven left permanent mandibular teeth were considered. Poor-quality radiographs exhibiting dark areas such as burnout, light areas, discrepancy in tooth size, superimposed structures, and those displaying artifacts were excluded. Radiographs documenting dental extractions or agenesis particularly in the lower left quadrant, evidence of systemic diseases, congenital anomalies, or genetic disorders, radiographs with the sign of any pathology in bone or dental tissues, presence of any cavity filling or endodontically treated permanent teeth, and radiographs of patients with ongoing orthodontic treatment were also excluded.

### 2.2. Study Sample

**Figure 1:**
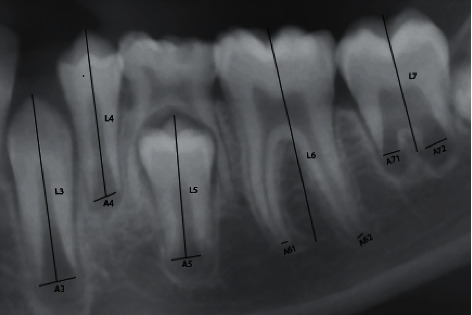
An example of Cameriere's measurements of mandibular teeth, *x*_*i*_ = *A*_*i*_/*L*_*i*_, *i* = 1,…, 7, of seven left mandibular teeth. *A*_*i*_, *i* = 1,…, 5 (teeth with one root), is the distance between the inner sides of the open apex. *A*_*i*_, *i* = 6, 7 (teeth with two roots), is the sum of the distances between the inner sides of the two open apices. *L*_*i*_, *i* = 1,…, 7, is the length of the tooth.

A total of 385 panoramic radiographs recorded between 2018 and 2020 were obtained from western states of Maharashtra, Gujarat, and Goa. Following inclusion and exclusion criteria, 67 panoramic radiographs were excluded from the research. The reasons for exclusion were hypodontia, impacted teeth, presence of cavity fillings and teeth that have undergone endodontic treatment in the lower left quadrant of jaws, and poor quality of radiographs. Seven panoramic radiographs were excluded owing to complete teeth development in those patients. The final sample thus consisted of 311 panoramic radiographs. Date of birth (DOB), date of radiographic examination (DOR), and gender were obtained from patient's medical records. The chronological age (CA) for each patient was calculated by subtracting the DOB from DOR using a simple formula in Microsoft Excel. Each panoramic radiograph was saved as a “jpg file” and processed using a computer-aided drafting program (Adobe Photoshop CS6). 311 panoramic radiographs were analysed according to the study conducted by Cameriere et al. [14]. Seven left lower permanent teeth, except wisdom teeth, were included for assessment. The number of teeth with closed apices and complete root development (*N*0) was calculated. In single-rooted teeth, the distance between the inner sides of the open apex (*A*_*i*_, *i* = 1,…, 5) was measured. In teeth with two roots, the sum of the distances between the inner sides of the two open apices (*A*_*i*_, *i* = 6, 7) was assessed (Figure 1). To rule out any possible differences in magnification and angulation among radiographs, the measurements were normalized by dividing with the tooth length (*L*_*i*_, *i* = 1,…, 7). Dental maturity was assessed using the normalized measurements of the seven left permanent mandibular teeth (*x*_*i*_ = *A*_*i*_/*L*_*i*_, *i* = 1,…, 7), the sum of the normalized open apices (*s*), and the number of teeth with complete root development (*N*0). Measurements were carried out by the two observers. Intra- and interobserver reproducibility was assessed by reexamining a random sample of 30 panoramic radiographs after a 2-week interval.

### 2.3. Statistical Analysis

For each individual, the morphological parameters, *x*_*i*_, *i* = 1,…, 7, *s*, N0, and subjects' gender, were entered in an Excel spreadsheet to use as predictive variables for estimating age in the following statistical analysis.

Using intraclass correlation coefficients, the repeatability and reproducibility of normalized widths of open apices or intraobserver and interobserver agreement was calculated. The intra- and interobserver agreement of those teeth with complete root development or *N*0 was calculated using Cohen's kappa score (kappa).

A multiple linear regression model with first-order interactions was developed to find an estimate of age as a function of the morphological variables. This was done by selecting those variables that contributed significantly to age estimation by using a stepwise selection method. A new age estimation formula (Western Indian formula-WIF) was created based on observed significant predictors of chronological age. Dental age was evaluated with the newly created Western Indian formula using Cameriere's method. The same set of panoramic radiographs was used to validate this formula. For each child, the real age was calculated as the difference between chronological age (CA) and dental age according to the Western Indian formula (DA) or CA-DA. The positive values of CA-DA indicated underestimation of dental age, while negative values showed overestimation.

Statistical analysis was performed using “Statistical Package for the Social Sciences” (SPSS Inc., released 2009, SPSS Statistics for Windows, version 17.0, Chicago: SPSS Inc.) software. The level of significance was set at 5%.

## 3. Results

The distribution of the panoramic radiographs according to age groups and gender is given in Table 1. The mean chronological ages were 10.38 ± 2.40 and 10.02 ± 2.78 years in males and females, respectively. There were no statistically significant intra- and interobserver differences observed between the paired sets of measurements which were carried out on the reexamined panoramic radiographs. Intraclass correlation coefficients for the same and a different observer were *r* = 0.997 (*P* < 0.001) and *r* = 0.923 (*P* < 0.001) which is in almost excellent agreement. In the seven left permanent mandibular teeth with complete root development (*N*0), no misfit was observed between the two measurements made by the same and a different observer, i.e., kappa = 1.

**Figure 2:**
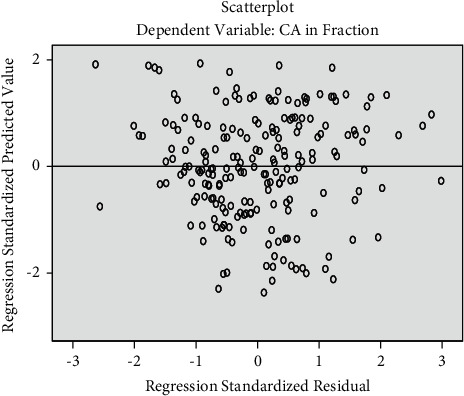
Residuals against the fitted values by using the regression model.

Figure 2 shows the residual scattered plot which is randomly dispersed around the horizontal axis which confirms the appropriateness of selecting the linear regression model for this study. Pearson's correlation coefficients between age and morphological variables revealed a significant correlation of age and the following variables, gender, variable *x*_5_ (second premolar), *s*, and *N*0 (Table 2). Thus, inclusion of these variables in the regression model generated the following linear regression formula:

(1) $$\begin{array}{c} \text{age}=11.664-2.806\left(x_{5}\right)+0.602\left(N0\right)-0.487\left(s\right)-0.819\left(\text{gender}\right), \end{array}$$

where “g” is 1 for males and 2 for females.

Statistical analysis revealed that all the variables that were significant predictors of age for the European population were the same in the Western Indian population. Equation (1), with the above considered variables, described a total deviance of *R* = 85% (*R*^2^ = 0.722). The median of the residuals (observed age minus predicted age) was −0.17 years, with an interquartile range of 0.74 years.

**Figure 3:**
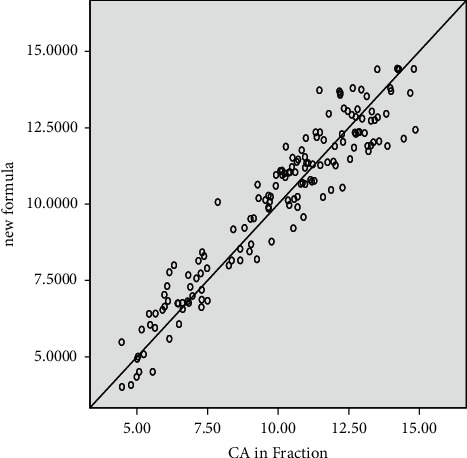
Plots of the chronological against estimated age in males.

**Figure 4:**
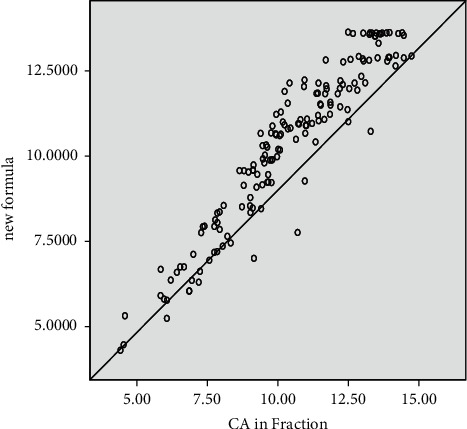
Plots of the chronological against estimated age in females.

The scattered plot (Figures 3 and 4) for both genders shows a tendency to cluster towards the middle of the plot. The regression model fits the trend of the data as the scattered plot (Figures 3 and 4) of the residuals against the fitted values did not show any obvious pattern. Mean differences and standard deviation in years between the dental and chronological age for age cohorts are presented for females in Table 3 and for males in Table 4.

### 3.1. Test of Residual Normalcy

**Figure 5:**
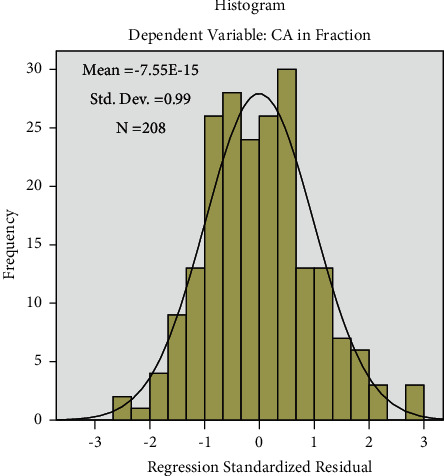
Histogram of the residuals against the fitted values by using the regression model.

The histogram (Figure 5) of the residual is used to check whether the variance is normally distributed. It shows a symmetrical bell-shaped curve which is evenly distributed around zero indicating normality in random error.

## 4. Discussion

The Study Group on Forensic Age Diagnostics (AGFAD; https://agfad.uni-muenster.de) [16] describes three key characteristics in estimating chronological age. They are physical examination, dentition status, and radiographic examination of the dentition and radiographic examination of the left hand [17]. Dental maturity estimation plays a vital role in the identification of delayed or advanced maturation. Stages of tooth development with morphological changes of the tooth crown and root have been widely used for assessing chronological age. Dental structures are less influenced by genetic and environmental factors than skeletal structures; therefore, using them as an aid for age estimation is more practical. Thus, it enables proper treatment planning and helps us understand the growth of permanent teeth [18]. It is therefore essential to perform dental maturity assessment as precisely as possible.

Several odontological methods are performed to determine age and assess phases of eruption within acceptable error limits. Such methods essentially define the stages of tooth mineralization which are seen on radiographs and coded based on predetermined scores.

The approach for dental age estimation proposed by Cameriere et al. is one of the techniques used in forensics, anthropology, clinical orthodontics, and pediatric dentistry [14]. In this method, a mathematical equation is used which is derived from linear regression analysis. It uses different tooth measurements to calculate chronological age as independent variables. This method of age assessment is an attempt to make age estimation more precise and reliable by taking into account other dental features and comparing different statistical models [14]. It has been tested worldwide with excellent results. Several studies have shown that dental growth and development are influenced by a specific population [15, 19–23]. The population-specific Indian formula by Rai et al. and Sharma et al. reported 89.7% and 85.6% of the total variation in chronological age [15, 24]. A similar study in the North Chinese population revealed a more significant coefficient of determination (*R* = 91.2%) when the population-specific formula was used [19]. Our study explains *R* = 85% of total variance (*R*^2^ = 0.72). The results indicate the suitability of the sum of normalized open apices (*s*), the number (*N*0) of teeth with complete root development, the variable *x*_5_ (second premolar), and gender as predictive markers. Thus, it is evident from most studies that the development of new population-specific formulae yielded better results.

In the present study, panoramic radiographs within the age range of 4 and 12 years revealed no significant difference in chronological age and dental age for both genders, which suggests that the formula precisely predicts chronological age amongst the Western Indian population. Thus, in this study, the WIF was found to be more appropriate for the Western Indian sample in this age group. The greatest underestimation was found in the age group of 14-15 years in both sexes. The mean values of CA-DA (WIF) in the age group of 14-15-year-old males and females were 0.74 ± 1.08 years and 1.21 ± .43 years, respectively. In addition to the 14-year age group, underestimation of dental age was observed amongst males in the age group of 13-14 years (0.77 ± 0.76). Latic-Dautovic et al. reported a similar error in the 14-year age group when they used the Cameriere European formula on the sample panoramic radiographs of orthodontic patients in Bosnia and Herzegovina [25]. A recent meta-analysis by Hostiuc et al. evaluated the actual variability of the mean difference between the chronological and dental age using the Cameriere method in different age groups. This meta-analysis revealed higher chronological ages compared to dental ages in older subadults which is in accordance with our study [26]. One of the reasons for the underestimation of age was difficulty in assessing the small apex opening, which is nearly closed or closed in older children [27]. Liversidge identified that only a few children at 13 years of age had immature second molars and were excluded from the testing sample. The proportion of children with incomplete maturation of the second molar seen on the panoramic radiographs and included in the study decreased considerably [18]. Ambarkova et al. reported that complete maturation of the second molars was first seen in children aged 12 years, whereas at the 13 years of age, 27 of 32 (84%) girls and 13 of 32 (41%) boys showed complete maturation of the second molar [28]. Therefore, the specific regression model and proportion of individuals with delayed maturation might be the reasons for the underestimation of dental age in the 13- and 14-year-old children. Further studies are required to address this matter extensively [29].

Statistical analysis revealed that all the variables that were significant predictors of age for the European population were the same in the Western Indian population. However, an Indian formula developed by Rai et al. on panoramic radiographs of individuals aged 3–15 years found the region variable and the first-order interaction between *s* and *N*0 as significant predictors of the formula. They added a dummy variable (*C* = 0 for Central and North India and *C* = 1 for South India) in the Indian formula to consider the impact of different social, ethnic, and nutritional factors in different parts of the country [15]. A recent formula for the North Indian population identified *x*_4_ (first premolar), *x*_1_ (central incisors), *x*_6_ (first molar), *s*, and *N*0 as significant predictors of the linear regression formula.

The differences in chronological ages and dental ages (irrespective of the method used) can be attributed to several factors such as precision of the method's execution, subjectivity of the examiner, sample size and structure, age, gender, nationality, ethnicity, and social status.

## 5. Conclusion

The current study shows that the WIF gave accurate results for the Western Indian population samples between 4 and 15 years of age. The results found in this study, like others, point out the population-specific influences of growth and development. Hence, we conclude that the open apex method of age evaluation by Cameriere et al. is very useful and can be recommended for applications both in clinical dentistry and forensic sciences in the Indian population.

## Figures and Tables

**Table 1:** Age and gender distribution of study subjects.

Chronological age range	Indian sample		
	Male (*N*)	Female (*N*)	Total (*N*)
4.00–4.99	4 (57.1%)	3 (42.9%)	7 (100%)
5.00–5.99	13 (81.3%)	3 (%18.8)	16 (100%)
6.00–6.99	15 (62.5%)	9 (37.5%)	24 (100%)
7.00–7.99	11 (42.3%)	15 (57.7%)	26 (100%)
8.00–8.99	7 (43.8)	9 (56.3%)	16 (100%)
9.00–9.99	15 (31.9%)	32 (68.1%)	47 (100%)
10.00–10.99	27 (55.1%)	22 (44.9%)	49 (100%)
11.00–11.99	17 (47.2%)	19 (52.8%)	36 (100%)
12.00–12.99	22 (57.9%)	16 (42.1%)	38 (100%)
14.00–14.99	8 (53.3%)	7 (46.7%)	15 (100%)
Total	154 (49.5%)	157 (50.5%)	311 (100%)

**Table 2:** Stepwise regression analysis predicting chronological age from the chosen predictors' coefficients (a).

Model		Unstandardized coefficients		Standardized coefficients	*t*	Sig.
		*B*	Std. error	Beta		
1	(Constant)	11.664	0.388		30.060	0.000
	*x*_5_	−2.806	0.678	−0.279	−4.138	0.000
	*N*0	0.602	0.076	0.435	7.921	0.000
	*s*	−0.487	0.168	−0.216	−2.897	0.004
	Gender	−0.819	0.122	−0.254	−6.703	0.000

a: dependent variable: CA in fraction.

**Table 3:** Mean values between the chronological age and the dental age (DA-CA) using the Western Indian formula (WIF) in females.

Age group	*n*	CA (mean ± SD)	DA (WIF) (mean ± SD)	CA-DA (WIF) (mean ± SD)	SEM	*t*	*P*
4.00–4.99	3	4.50 ± .081	4.70 ± 0.54	−0.019 ± 0.47	0.276	−0.692	0.560
5.00–5.99	3	5.88 ± 0.07	6.13 ± 0.47	−0.24 ± 0.51	0.299	−0.832	0.493
6.00–6.99	9	6.51 ± .34	6.21 ± 0.49	−0.29 ± 0.46	0.155	1.928	0.090
7.00–7.99	15	7.58 ± 0.31	7.57 ± 0.63	−0.00 ± 0.52	0.134	0.029	0.978
8.00–8.99	9	8.50 ± 0.34	8.59 ± 0.92	−0.08 ± 0.67	0.226	−0.385	0.710
9.00–9.99	32	9.50 ± 0.30	9.63 ± 0.88	−0.13 ± 0.72	0.12	−1.085	0.286
10.00–10.99	22	10.49 ± 0.34	10.83 ± 0.97	−0.33 ± 1.04	0.22	−1.512	0.145
11.00–11.99	19	11.51 ± 0.25	11.49 ± 0.55	−0.01 ± 0.49	0.113	0.122	0.904
12.00–12.99	16	12.49 ± 0.25	12.25 ± 0.74	0.24 ± 0.70	0.176	1.365	0.192
13.00–13.99	22	13.47 ± 0.31	13.14 ± 0.68	0.33 ± 0.68	0.146	2.291	0.032
14.00–14.99	7	14.38 ± 0.19	13.16 ± 0.40	1.21 ± 0.43	0.164	7.433	0.000

**Table 4:** Mean values between the chronological age and the dental age (DA-CA) using the Western Indian formula (WIF) in males.

Age group	SEM	CA (mean ± SD)	DA (WIF) (mean ± SD)	CA-DA (WIF) (mean ± SD)	SEM	*t*	*P*
4.00–4.99	4	4.67 ± 0.25	4.48 ± 0.68	−0.19 ± 0.81	0.40	−0.478	0.665
5.00–5.99	13	5.47 ± 0.35	5.76 ± 0.85	−0.29 ± .63	0.17	−1.684	0.118
6.00–6.99	15	6.51 ± 0.30	6.93 ± 0.62	−0.41 ± 0.68	0.17	−2.350	0.034
7.00–7.99	11	7.36 ± 0.20	7.78 ± 0.97	−0.42 ± 0.86	0.26	−1.634	0.133
8.00–8.99	7	8.59 ± 0.26	8.52 ± 0.49	0.06 ± 0.48	0.18	0.360	0.731
9.00–9.99	15	9.50 ± 0.30	9.83 ± 0.77	−0.32 ± 0.67	0.17	−1.890	0.080
10.00–10.99	27	10.57 ± 0.28	10.86 ± 0.71	−0.29 ± 0.75	0.14	−2.050	0.051
11.00–11.99	17	11.44 ± 0.28	11.57 ± 0.92	−0.12 ± 0.93	0.22	−0.529	0.604
12.00–12.99	24	12.51 ± 0.30	12.59 ± 0.87	0.07 ± 0.86	0.18	−0.398	0.695
13.00–13.99	15	13.43 ± 0.27	12.66 ± 0.79	0.77 ± 0.76	0.19	3.922	0.002
14.00–14.99	9	14.44 ± 0.31	13.69 ± 0.93	0.74 ± 1.08	0.38	1.949	0.092

## Data Availability

The data used to support the findings of this study are available from the corresponding author upon request.
